# Effects of different n-6/n-3 polyunsaturated fatty acids ratios on lipid metabolism in patients with hyperlipidemia: a randomized controlled clinical trial

**DOI:** 10.3389/fnut.2023.1166702

**Published:** 2023-06-01

**Authors:** Yiwei Yang, Yanping Xia, Baixi Zhang, Dan Li, Jiai Yan, Ju Yang, Jing Sun, Hong Cao, Yingyu Wang, Feng Zhang

**Affiliations:** ^1^Wuxi School of Medicine, Jiangnan University, Wuxi, China; ^2^Department of Nutrition, Affiliated Hospital of Jiangnan University, Wuxi, China; ^3^Clinical Evaluation Center for Functional Food, Affiliated Hospital of Jiangnan University, Wuxi, China; ^4^Yixing Institute of Food and Biotechnology Co., Ltd., Yixing, China; ^5^School of Food Science and Technology, Jiangnan University, Wuxi, China

**Keywords:** n-6/ n-3 polyunsaturated fatty acids ratio, cardiometabolic health, hyperlipidemia, lipid metabolism, perilla oil

## Abstract

**Background and aims:**

Intake of n-3 polyunsaturated fatty acids (PUFA) is helpful for cardiometabolic health. It improves lipid metabolism, and increasing n-3 PUFA is often considered beneficial. However, the role of n-6/n-3 in the regulation of lipid metabolism has been much debated. Therefore, this study was performed on the effect of different proportions of n-6/n-3 diet on lipid metabolism, and quality of life in patients with hyperlipidemia, aiming to explore appropriate proportions of n-6/n-3 to provide the theoretical basis for the development and application of nutritional blended oil in the future.

**Methods:**

These 75 participants were randomized and assigned into three groups, which received dietary oil with high n-6/n-3 PUFA ratios (HP group: n-6/n-3 = 7.5/1), dietary oil with middle n-6/n-3 PUFA ratios (MP group: n-6/n-3 = 2.5/1) or low n-6/n-3 PUFA ratios (LP group: n-6/n-3 = 1/2.5). All patients received dietary guidance and health education were monitored for hyperlipidemia. Anthropometric, lipid and blood glucose parameters and quality of life were assessed at baseline and 60 days after intervention.

**Result:**

After 60 days, high-density lipoprotein cholesterol (HDL-c) level was increased (*p* = 0.029) and Total cholesterol (TC) level was decreased (*p* = 0.003) in the MP group. In the LP group, TC level was decreased (*p* = 0.001), TG level was decreased (*p* = 0.001), but HDL-c level was not significantly increased. At the end of intervention, quality of life’ score was improved in both MP and LP groups (*p* = 0.037).

**Conclusion:**

Decreasing the intake of edible oil n-6/n-3 ratio can improve blood lipids and quality of life. This is significant for the prevention of cardiovascular disease (CVD). It is also essential to note that an excessive reduction of the n-6/n-3 ratio does not further improve the blood lipid metabolism. In addition, the application of perilla oil in nutritional blended oil has particular significance.

**Clinical trial registration:**

https://www.chictr.org.cn/indexEN.html, identifier ChiCTR-2300068198.

## 1. Introduction

Hyperlipidemia, also known as dyslipidemia (1, 2), is an essential risk factor leading to cardiovascular disease (CVD), including atherosclerosis and ischemic cerebrovascular accidents (3, 4). Hyperlipidemia has become one of the most common health problems in humans (5, 6), which seriously affects the quality of life of patients. Nevertheless, high-fat diets are commonly regarded as contributing factors to excess body fat accumulation. Moreover, excessive fat intake, particularly when derived from saturated fatty acids (SFAs), has been linked to an elevated risk of obesity and hyperlipidemia.

Dietary fats encompass a diverse array of fatty acids (FA), derived from various sources, each exhibiting distinct chemical structures and biological functions (7, 8). Numerous studies have demonstrated that dietary polyunsaturated fatty acids (PUFA) intake is associated with positive effects on obesity and CVD (9, 10). Among PUFAs, n-3 PUFAs and n-6 PUFAs play particularly significant biological roles. N-3 PUFAs including docosahexaenoic acid (DHA), eicosapentaenoic acid (EPA), alpha-linolenic acid (ALA), possess neuroprotective properties, reduce the expression of eicosane, pro-inflammatory factors, modulate other inflammatory mediators, and suppress inflammatory responses (14–16). Primary sources of ALA include perilla and flaxseed oils. Prior research has indicated that n-3 PUFA-rich foods or n-3 supplements can decrease body weight and mitigate the risk of CVD stemming from dyslipidemia, such as coronary atherosclerosis (11–13). However, it has also been reported that no improvement in disease risk with n-3 PUFA intake (14, 15). N-6 PUFAs, primarily composed of linoleic acid (LA) and arachidonic acid (AA), can be found in edible oils like peanut oil and soybean oil, as well as animal-derived foods. As an EPA precursor, LA facilitates inflammatory processes and stimulates immune responses, thereby exacerbating inflammatory damage (16). Research on n-6 PUFAs has shown that their consumption in a typical diet reduces both good and bad cholesterol levels. Furthermore, n-6 and n-3 PUFAs cannot be interconverted within the body. These PUFAs compete for desaturase and *carbon-chain elongase*, with the conversion of LA to AA, and ALA to EPA and DHA mediated by desaturation and elongation reactions. An increase in one type of PUFA intake results in a decreased in the synthesis of the other PUFA in the body (17). An imbalance in the dietary n-6/n-3 PUFA ratio can leads to chronic low-grade inflammatory responses, promoting inflammation (18). The development of dyslipidemia or CVD is associated with inflammation. Thus, emphasizing an importance of maintaining an optimal n-6 and n-3 PUFA ratio for overall health (19).

Dietary therapy, as an effective alternative measure to control dyslipidemia, is currently attracting increasing attention from experts and scholars, including the use of dietary oils (20, 21). Studies of dietary intervention clinical trials focusing on hypercholesterolemic subjects revealed that plasma triglycerides (TG) may be reduced through n-3 PUFA and supplementation or used the oil blend with the higher concentration of n-3 PUFA, while also exhibiting a trend toward decreased total and LDL cholesterol (LDL-c) (22). This highlights the significance of n-3 PUFAs in reducing the risk of CVD, as extensively investigated in previous research (15). *Perilla frutescens* (L.) Britt. (PF) is a prominent oilseed crop utilized as a source of edible vegetable oil, traditionally considered a health food in Asian countries such as Korea, China, and Japan (23, 24). In comparison to other edible vegetable oils, perilla seed oil (PO) is primarily consists of PUFA (76 ~ 93%), ALA(57 ~ 62%) and LA(14 ~ 18%) (25), with ALA, an essential n-3 PUFA, being the most abundant (26). Past studies have demonstrated that perilla oil exhibits beneficial effects on weight loss, decreased fat mass (FM) reduction, waist circumference (WC) reduction, and appetite responses is improvement, and lipid profile enhancement (27, 28). Consequently, the properties of perilla oil have been suggested for use in the treatment and prevention of obesity (29).

We hypothesized that a reduced n-6/n-3 PUFA ratio might mitigate some of the detrimental effects of dyslipidemia on cardiovascular function. Therefore, the aim of this study was to evaluate the effects of n-6/n-3 PUFA plant blend oils prepared with different proportions of soybean oil and perilla oil on body weight, blood lipids and blood glucose in middle-aged and older patients with hyperlipidemia.

## 2. Materials and methods

### 2.1. Subjects and study design

This study was a double-blind, randomized, placebo-controlled trial conducted in the Third Affiliated Hospital of Nantong University (later renamed the Affiliated Hospital of Jiangnan University) from April 2022 to October 2022 for 6 months. This trial was approved by the Ethics Committee of the Affiliated Hospital of Jiangnan University (ID: LS2021010) and registered in the Chinese Clinical Trial Registry with registration (ChiCTR-2,300,068,198). Written informed consent was obtained from all participants by completing a consent form before the intervention.

Seventy-five hyperlipidemic patients, with body mass index (BMI) between 28 and 34.9 kg/m^2^, aged 45 to 75 years, diagnosed with dyslipidemia and met any of the following criteria: TC ≥ 6.2 mmol/L, TG ≥ 2.3 mmol/L, HDL-c < 1.0 mmol/L, LDL-c ≥ 4.1 mmol/L. Furthermore, without a diagnosis of neurological, malignant hypertension, cancer or gastrointestinal disease, unrestricted diet, and no use of dietary supplements, herbal medicines, or lipid-lowering drugs in the last 2 months. Subjects with poor compliance or protocol violation or unwillingness to continue the clinical trial were asked to withdraw from this study. In the end, seventy-one participants were eligible and decided to participate in this study and completed. All subjects were randomly assigned to the control group (HP group) and the experimental groups (MP group, LP group) at a ratio of 1:1:1. The demographic data of all subjects were recorded after enrollment, including gender, age, disease history, medication, and lifestyle. Anthropometric data were collected, including BMI, waist circumference (WC), hip circumference (HC), waist-hip ratio (WHR), etc. Based on previous studies (30), we calculated the sample size based on the primary outcome. Considering 20% drop-out and nonadherence, a total of 75 study subjects were required for the HP, MP, and LP groups at a 1:1:1 ratio. All eligible participants were asked to complete a 3-day dietary record and maintain physical activity. Dietitians questioned and recorded the participants’ diets during the first 3 days of the formal experiment. Refer to the Chinese food composition table’s standard edition to better understand the participants’ diets. At the first enrollment, volunteers arrived at the Affiliated Hospital of Jiangnan University after a 12-h fast for blood collection and anthropometric assessment. Participants who met the diagnostic criteria of dyslipidemia were enrolled in the study and were given the corresponding cooking oil according to the random sequence. Participants were asked to replace the daily cooking oil at home and use perilla oil for cooking every day. The amount of oil used per person was 25 g per day, and they followed the prescribed dietary plan for 60 days.

The clinical trial was double-blinded because neither the subjects nor researchers knew which group every subject was assigned to, and the grouping of all subjects was unblinded by a statistician after they had completed their experiment. In the middle of the trial, volunteers were given oil for the next stage. Intervention adherence was assessed by perilla oil use diary.

### 2.2. Intervention

In this part, the study intervention will be described in detail. All participants were randomly assigned (allocation ratio: 1:1:1) to groups HP, MP and LP. In both the control and experimental groups, we administered the oil at a dose of 25 g per day, and all subjects received the same dose of oil. In the HP group, subjects received soybean oil, while the MP and LP groups received perilla blend oil with n-6/n-3 = 2.5/1 and n-6/n-3 = 1/2.5, respectively (Tables 1, 2). The duration of the intervention was 60 days. The soybean oil used in the study was extracted from soybeans and produced by the Huanan Nongshengyuan Food Co., Ltd. of China Longjiang Forest Industry General Company. Perilla oil was extracted from perilla seed and supplied by the Huanan Nongshengyuan Food Co., Ltd. of China Longjiang Forest Industry General Company. Both oils were cold-pressed and stored in identical black bottles. Both oils were kept in the shade according to the manufacturer’s instructions. During the intervention, participants were instructed to not change their dietary habits and physical activity. Importantly, the doses of applied oils were exchanged for the same amount of oil used in the diet. Therefore, the energy contents of the diet did not change during the intervention. Moreover, during the intervention, subjects were supervised over the telephone by the nutritionist to check compliance with the study protocol, especially regular administration of the appropriate oil. To verify adherence to the study protocol, participants were instructed to regularly return empty oil bottles to the research team. No deviation from the study protocol was noted.

**Table 1:** Qualitative analysis of fatty acids in perilla oil and soybean oil.

Fatty acids (FA)		Perilla oil	Soybean oil
Palmitic acid (C16:0)	%(A/A)	6.04	10.88
Stearic acid (C18:0)	%(A/A)	1.67	4.56
Arachidic acid (C20:0)	%(A/A)	ND	0.45
Behenic acid (C22:0)	%(A/A)	ND	0.47
Lignoceric acid (C24:0)	%(A/A)	ND	0.18
Oleic acid (C16:1, 9c)	%(A/A)	ND	0.09
Oleic acid (C18:1, 9c)	%(A/A)	11.8	24.27
Cis-vaccenic acid (C18:1, 11c)	%(A/A)	ND	0.04
Eicosenoic acid (C20:1)	%(A/A)	0.12	0.23
Linoleic acid (C18:2, 9c, 12c, n-6)	%(A/A)	13.5	52.49
α-linolenic acid (C18:3, 9c,12c, 15c n-3)	%(A/A)	66.4	5.99
Total saturated FA(SFA)	%(A/A)	1.67	16.54
Total monounsaturated FA (MUFA)	%(A/A)	11.92	24.63
Total polyunsaturated FA(PUFA)	%(A/A)	79.9	58.54
Total	%(A/A)	99.53	99.71

ND, not detect.

**Table 2:** Ratio of soybean oil to perilla oil.

Group	Soybean oil: perilla seed oil	LA (%)	ALA (%)	n-6: n-3 ratio
HP	25:0	13	1.7	7.5:1
MP	20:5	10.9	4.7	2.5:1
LP	5:20	5.2	13.6	1:2.5

HP group: High proportion of n6/n3 group (n6/n3 = 7.5/1). MP group: Middle proportion of n6/n3 group (n6/n3 = 2.5/1). LP group: Low proportion of n6/n3 group (n6/n3 = 1/2.5).

In addition, health education for patients by endocrinology nurses, mainly including improving the understanding of dyslipidemia, correcting misconceptions about the cause of illness (i.e., providing information about the nature of the disease and that dyslipidemia is multifactorial, has multiple effects, and is often related to genetics and poor daily living habits), and reducing their fear of hyperlipidemia and its complications. Telephone-based Health follow-up service, asked about participant’s daily, weekly, and monthly oil consumption, and received telephone guidance on daily living and exercise.

### 2.3. Anthropometric evaluation

Anthropometry and body weight was measured by an electronic weight scale with 150 kg of capacity and 100 g of precision, and height was measured by a vertical rangefinder measured height. Both variables were used to calculate BMI. WC was measured at the midpoint between the last rib and the top edge of the iliac crest, and HC was measured in the largest area of the hip to evaluate the WHR. WC higher than 102 cm was considered a risk of metabolic complications associated with obesity, and WHR higher than 0.90 was considered a cardiovascular risk. According to the principle that different biological tissues and organs have different electrical properties. Body fat measuring instrument (Biospace Inbody270, Biospace Corporation, Korea) is used to measure the resistance of different parts of the human body. The composition information of the corresponding parts is analyzed, such as fat, muscle mass, protein, inorganic salts, water, and so on.

### 2.4. Biochemical evaluation

The primary outcome of lipid change was measured before and after the intervention. Lipid metabolism indicators included total cholesterol (TC), triglyceride (TG), high-density lipoprotein cholesterol (HDL-c), and low-density lipoprotein cholesterol (LDL-c). Secondary outcomes included changes in fasting plasma glucose (FBG) and uric acid level (UA). The above indicators were tested by an automatic biochemical analyzer (Beckman Coulter au680; Beckman Coulter Corporation, America).

### 2.5. Quality of life measurement

The MOS 36-Item Short Form Health Survey (SF-36) was used to measure the improvement of the quality of life of the study subjects. SF-36 is a concise health questionnaire developed by the Boston Institute of Health Research (31). It is widely used in the measurement of quality of life in the general population, the evaluation of clinical trials, and the evaluation of health policies. As a simple health questionnaire, SF-36 comprehensively summarizes the quality of life of the respondents from eight aspects: physical functioning, role physical, bodily pain, general health, vitality, social functioning, role emotional, and mental health. In addition to the eight components of the SF-36, a health change measure was also included to assess the overall change in health status over the past year (32). The SF-36 scale has good reliability and validity, and its application range is wide, which is suitable for the evaluation of the health level of Chinese residents.

### 2.6. Statistical analysis

Statistical analysis was performed using SPSS software version 26.0 (SPSS Inc., Chicago, IL, USA), considering significant *p* < 0.05. Shapiro Wilks (normally). Test was performed to evaluate the distribution of the variables. Differences among the three tested groups at baseline and at the end of the study were assessed by one-way ANOVA test or Kruskal-Wallis test. When within-group comparison before and after intervention and between-group comparison, the distribution was normal being used the Student’s *t* test and non-normal being used the Wilcoxon or Mann–Whitney test. Quality of life scales were analyzed by covariance analysis.

## 3. Results

### 3.1. Study population

**Figure 1:**
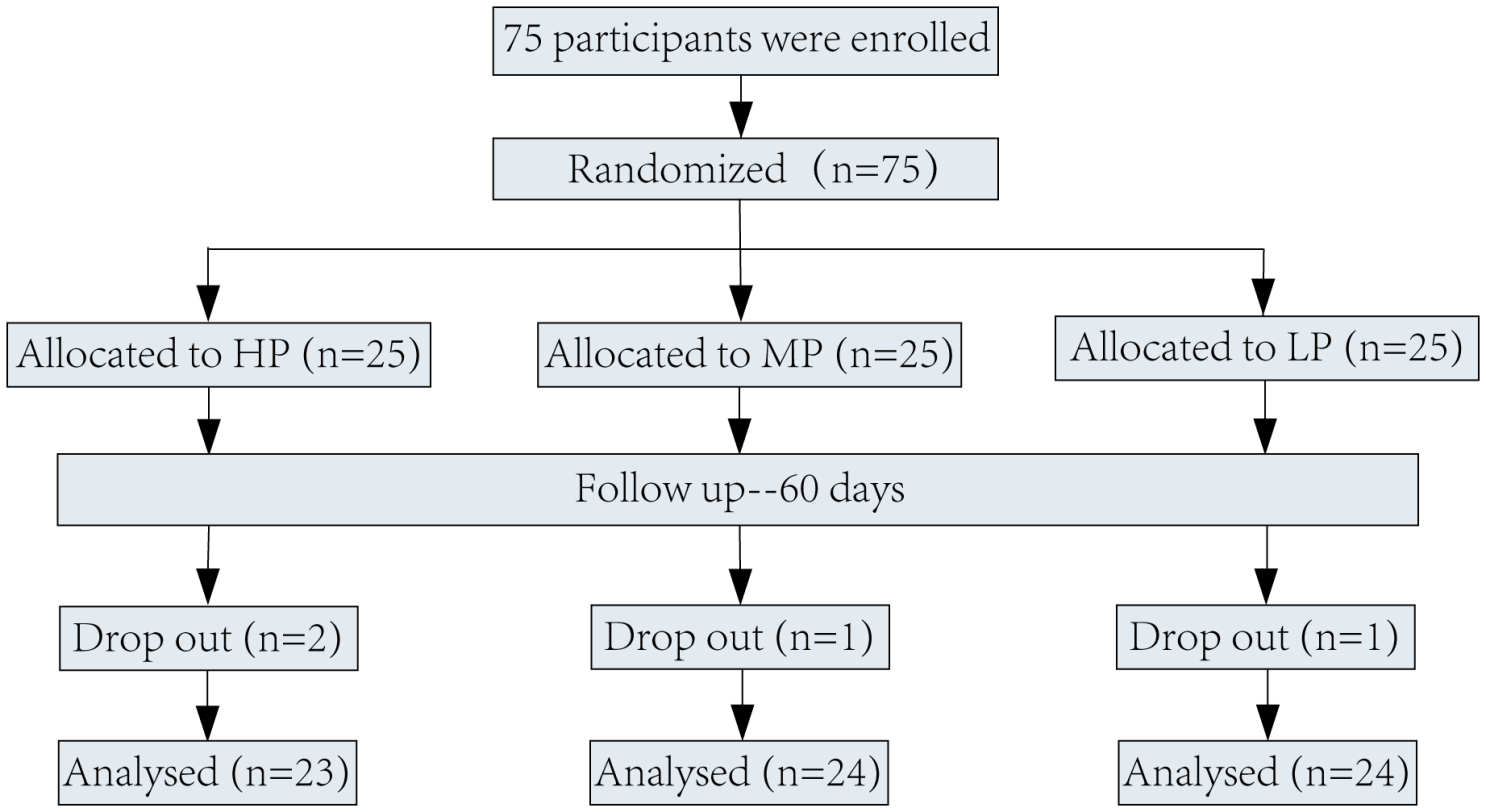
Flow-diagram of volunteers of the study. HP group: High proportion of n6/n3 group (n6/n3 = 7.5/1). MP group: Middle proportion of n6/n3 group (n6/n3 = 2.5/1). LP group: Low proportion of n6/n3 group (n6/n3 = 1/2.5).

The participant flowchart is presented in Figure 1. During the intervention period, two participants dropped out of the HP group, one out of the MP group and one out of the LP group. Finally, a total of 71 patients with hyperlipidemia completed the study.

### 3.2. Anthropometric evaluation

The baseline characteristics are presented in Table 3. All parameters of participants were similar between the three groups at baseline. Diet with a lower n-6/n-3 PUFA ratio did not change body fat or WHR of the participants after intervention. Body weight and BMI decreased in three groups, as shown in Table 3. The delta values of body weight and BMI of each group were summarized as follow: Δ Body weight-HP group: −0.93 ± 4.44, MP group: −1.44 ± 3.97, LP group: −2.84 ± 5.88, *p =* 0.376; Δ BMI-HP group: −0.34 ± 1.9, MP group: −0.57 ± 1.53, LP group: −1.12 ± 2.36, *p* = 0.386).

**Table 3:** Baseline characteristics of study participants.

Variables		HP (*n* = 23)	Δ	MP (*n* = 24)	Δ	LP (*n* = 24)	Δ	Value of *p*
Sex (male/female)		10/13		11/13		10/14		0.958
Age (years)		58.13 ± 6.73		60.88 ± 5.44		60.08 ± 5.59		0.273
Height (m)		162.22 ± 6.91		160.71 ± 6.86		161.28 ± 5.37		0.629
Anthropometry								
Body Weight (kg)	Baseline	78.96 ± 6.58		76.91 ± 8.63		77.93 ± 6.17		0.691
	Final	78.03 ± 7.00	−0.93 ± 4.44	75.47 ± 8.54	−1.44 ± 3.97	75.09 ± 6.12	−2.84 ± 5.88	0.500
	Value of *p*	0.328*		0.088*		0.027*		0.376^#^
BMI (kg/m2)	Baseline	30.03 ± 2.10		29.75 ± 2.40		30.10 ± 2.12		0.810
	Final	29.65 ± 1.94	−0.34 ± 1.69	29.18 ± 2.24	−0.57 ± 1.53	28.97 ± 1.81	−1.12 ± 2.36	0.500
	Value of *p*	0.294*		0.083*		0.030*		0.386^##^
WHR	Baseline	0.873 ± 0.036		0.875 ± 0.036		0.873 ± 0.034		0.892
	Final	0.867 ± 0.040	−0.006 ± 0.019	0.870 ± 0.044	−0.005 ± 0.026	0.872 ± 0.041	−0.001 ± 0.024	0.913
	Value of *p*	0.144*		0.341*		0.799*		0.754^##^
Body fat (%)	Baseline	27.23 ± 3.18		28.22 ± 2.76		27.28 ± 2.62		0.424
	Final	27.37 ± 2.57	0.12 ± 1.80	27.23 ± 2.52	−0.99 ± 1.72	26.45 ± 2.34	−0.83 ± 1.993	0.393
	Value of *p*	0.749*		0.010*		0.054*		0.093^##^

HP group: High proportion of n6/n3 group (n6/n3 = 7.5/1). MP group: Middle proportion of n6/n3 group (n6/n3 = 2.5/1). LP group: Low proportion of n6/n3 group (n6/n3 = 1/2.5). BMI: body mass index. WC: waist circumference. WHR: waist-hip ratio. Δ: delta (final value-after intervention value). * Student’s *t* test, paired. ^#^ One-way ANOVA. ^##^ Kruskal-Wallis.

### 3.3. Blood lipid parameters

There were significant differences in the mean values for partial outcomes between groups at the end of the dietary intervention (Table 4). The dietary intervention with perilla oil treatment was effective in improving the serum levels of TC (*p* = 0.002) and TG (*p* = 0.006). A declining trend was observed for LDL-c levels in the MP group and LP group, although the difference was not significant.

**Table 4:** Multiple comparisons of the mean values at the end of follow up in all intervention groups in individuals with patients.

Endpoints at the end of follow up	All groups (*n* = 71)	HP vs. MP	HP vs. LP	MP vs. LP
	Value of *p*	Value of *p*	Value of *p*	Value of *p*
Body Weight (kg)	0.500*	0.430**	0.431**	0.854**
BMI (kg/m2)	0.500*	0.670**	0.580**	0.720**
WHR	0.913*	0.971**	0.964**	0.970**
Body fat (%)	0.393*	0.845**	0.503**	0.504**
Serum TC (mmol/L)	0.002*	0.004**	0.012**	0.580**
Serum TG (mmol/L)	0.006^#^	0.005^##^	0.098^##^	0.192^##^
Serum HDL-c (mmol/L)	0.109*	0.131**	0.632**	0.227**
Serum LDL-c (mmol/L)	0.165^#^	0.219^##^	0.651^##^	0.334^##^
Plasma glucose (mmol/L)	0.769*	0.868**	0.879**	0.868**
Plasma uric acid (mmol/L)	0.330^#^	0.362^##^	0.656^##^	0.656^##^

HP group: High proportion of n6/n3 group (n6/n3 = 7.5/1). MP group: Middle proportion of n6/n3 group (n6/n3 = 2.5/1). LP group: Low proportion of n6/n3 group (n6/n3 = 1/2.5). BMI: body mass index. WC: waist circumference. WHR: waist-hip ratio. the data was showed as mean ± standard deviation. * One-way ANOVA. ^#^ Kruskal-Wallis Test. ** Student’s t test, unpaired. ^##^ Mann–Whitney.

The results of the three groups before and after intervention were shown in Table 5. After the end of the intervention, HDL-c levels in the MP group increased from 1.313 to 1.500 mmol/L (*p* = 0.029). The difference in the TC, TG and LDL-c levels before and after intervention was statistically significant (*p* < 0.05, paired *T*-test). LP group showed decreased TC levels (6.711 ± 1.104 vs. 5.843 ± 0.596, *p* = 0.001) and TG: (2.332 ± 0.737 vs. 1.741 ± 0.617, *p* = 0.001) with no change in HDL levels before and after intervention. In addition, as shown in Table 5, all differences of ΔTC, ΔTG and ΔHDL-c between the three groups were statistically significant (ΔTC: *p* = 0.023, ΔTG: *p* = 0.003, ΔHDL-c: *p* = 0.002). Despite not being statistically significant, a clear reduction in LDL-c in HP and LP group were observed: ΔLDL-c = −0.171 ± 0.395 (HP group) and ΔLDL-c = −0.230 ± 0.888 (LP group).

**Table 5:** Multiple comparisons of the mean values at the end of follow up in all intervention groups in individuals with patients.

Blood biochemistry		HP (*n* = 23)	Δ	MP (*n* = 24)	Δ	LP (*n* = 24)	Δ	Value of *p*
Serum TC (mmol/L)	Baseline	6.567 ± 0.914		6.124 ± 0.889		6.711 ± 1.104		0.220
	Final	6.562 ± 1.064	−0.004 ± 1.042	5.703 ± 0.887	5.703 ± 0.887	5.843 ± 0.596	−0.868 ± 1.140	0.002
	Value of *p*	0.984*		0.041*		0.001*		0.023^#^
Serum TG (mmol/L)	Baseline	2.111 ± 0.827		1.933 ± 1.019		2.332 ± 0.737		0.186
	Final	2.169 ± 0.834	0.058 ± 0.694	1.461 ± 0.748	1.461 ± 0.748	1.741 ± 0.617	−0.591 ± 0.573	0.006
	Value of *p*	0.691*		0.003**		0.001**		0.003^##^
Serum HDL (mmol/L)	Baseline	1.433 ± 0.328		1.433 ± 0.328		1.391 ± 0.308		0.389
	Final	1.316 ± 0.313	−0.117 ± 0.253	1.500 ± 0.254	1.500 ± 0.254	1.360 ± 0.354	−0.031 ± 0.211	0.109
	Value of *p*	0.038*		0.029*		0.476*		0.002^#^
Serum LDL (mmol/L)	Baseline	4.137 ± 0.685		3.832 ± 0.758		4.180 ± 0.880		0.356
	Final	4.180 ± 0.880	−0.083 ± 0.500	3.661 ± 0.744	3.661 ± 0.744	3.950 ± 0.788	−0.230 ± 0.888	0.165
	Value of *p*	0.406*		0.045**		0.218**		0.745^#^
Plasma glucose (mmol/L)	Baseline	5.66 ± 0.66		5.69 ± 0.55		5.85 ± 0.51		0.318
	Final	5.80 ± 0.57	0.14 ± 0.45	5.72 ± 0.58	5.72 ± 0.58	5.83 ± 0.53	−0.03 ± 0.23	0.769
	Value of *p*	0.159*		0.759*		0.572*		0.329^#^
Plasma uric acid (mmol/L)	Baseline	314.46 ± 26.49		300.40 ± 25.37		307.28 ± 27.66		0.116
	Final	315.03 ± 23.45	0.57 ± 29.67	303.32 ± 30.92	303.32 ± 30.92	309.68 ± 25.31	2.41 ± 20.41	0.330
	Value of *p*	0.927**		0.425*		0.569*		0.935^##^

HP group: High proportion of n6/n3 group (n6/n3 = 7.5/1). MP group: Middle proportion of n6/n3 group (n6/n3 = 2.5/1). LP group: Low proportion of n6/n3 group (n6/n3 = 1/2.5). Δ: delta (final value – after intervention value). *Student’s *t* test, paired. **Wilcoxon test. ^#^One-way ANOVA. ^##^Kruskal-Wallis.

### 3.4. The SF-36 quality of life score

Quality of life scores among the three groups before and after intervention were shown in Table 6. Perilla oil intervention had significantly different effects on vitality scores (HP vs. MP vs. LP): (66.09 ± 9.648 vs.69.17 ± 7.470 vs. 68.96 ± 7.799, *p* = 0.008) and the total scores (68.841 ± 4.586 vs. 70.613 ± 4.817 vs. 71.277 ± 4.195, *p* = 0.037) had significant difference. After the intervention, the MP group’s scores had significant differences in vitality, social functioning, and total score compared with pre-intervention (VT: 63.13 ± 9.066 vs. 69.17 ± 7.470, *p* = 0.001, SF: 69.29 ± 11.686 vs. 2.96 ± 9.720, *p* = 0.029, Total score: 67.034 ± 6.899 vs. 70.613 ± 4.817, *p* = 0.009). Additionally, the scores of LP group had significant differences in role-physical, role-emotional, mental health, and total score compared with pre-intervention (RP: 61.46 ± 28.532 vs. 69.79 ± 20.824, *p* = 0.029, VT: 61.67 ± 8.427 vs. 68.96 ± 7.799, *p* = 0.000, MH: 60.67 ± 10.277 vs. 64.42 ± 10.450, *p* = 0.001, Total score:67.411 ± 6.283 vs.71.277 ± 4.195, *p* = 0.001).

**Table 6:** Quality of life scores among the three groups before and after the intervention.

SF-36	HP (*n* = 23)		MP (*n* = 24)		LP (*n* = 24)		*F*^a^	*p*-value
	Pre	Post	Pre	Post	Pre	Post		
PF	85.43 ± 4.980	85.65 ± 4.599	86.46 ± 4.773	87.29 ± 3.895	87.71 ± 4.165	88.13 ± 3.555	1.189	0.331
RP	63.04 ± 27.041	67.39 ± 17.573	60.42 ± 25.449	67.71 ± 18.765	61.46 ± 28.532	69.79 ± 20.824*	0.371	0.692
BP	64.65 ± 19.676	63.96 ± 18.656	65.46 ± 19.204	68.92 ± 14.407	64.92 ± 20.151	66.50 ± 17.230	0.899	0.412
GH	55.978 ± 17.524	57.337 ± 16.499	58.854 ± 18.237	60.156 ± 13.266	59.375 ± 15.309	59.635 ± 12.766	0.078	0.925
VT	65.43 ± 9.404	66.09 ± 9.648	63.13 ± 9.066	69.17 ± 7.470*	61.67 ± 8.427	68.96 ± 7.799*	5.231	0.008
SF	70.83 ± 13.917	71.78 ± 11.878	69.29 ± 11.686	72.96 ± 9.720*	71.13 ± 13.300	73.42 ± 9.127	0.811	0.419
RE	72.65 ± 19.201	74.17 ± 13.917	71.00 ± 20.449	75.25 ± 14.597	72.38 ± 21.262	79.38 ± 16.320	1.045	0.357
MH	63.86 ± 9.064	64.35 ± 9.335	61.67 ± 10.277	63.67 ± 9.649	60.67 ± 10.277	64.42 ± 10.450*	1.699	0.191
Total	67.731 ± 4.944	68.841 ± 4.586	67.034 ± 6.899	70.613 ± 4.817*	67.411 ± 6.283	71.277 ± 4.195*	3.452	0.037

HP group: High proportion of n6/n3 group (n6/n3 = 7.5/1). MP group: Middle proportion of n6/n3 group (n6/n3 = 2.5/1). LP group: Low proportion of n6/n3 group (n6/n3 = 1/2.5). PF: Physical Functioning. RP: Role-Physical. BP: Bodily Pain. GH: General Health. VT: Vitality. SF: Social Functioning. RE: Role-Emotional. MH: Mental Health. Value of *p*: Analysis of covariance; *Compared with the group pre-intervention, *p* < 0.05.

## 4. Discussion

Hyperlipidemia, a significant causal risk factor for CVD, is a chronic condition linked to metabolic complications (33). Effective interventions are crucial for treating and preventing dyslipidemia and its associated complications (34). Dietary intervention is regarded as an efficacious and safe treatment approach. In this context, we examined the hypothesis that a diet with a lower n-6/n-3 ratio would promote weight loss and improved lipid levels. Our finding revealed that the diet characterized by a reduced n-6/n-3 ratio led to increased HDL-c level and decreased TC, TG and LDL-c levels in hyperlipidemic patients. In the present study, we observed a decrease in LDL level in the MP group, while HDL levels increased following perilla oil consumption. Elevated HDL levels facilitate the reverse transport of cholesterol from peripheral tissues to the liver for more efficient utilization. As PUFAs intake increases, there is a corresponding rise in HDL synthesis to facilitate this reverse transport. Although limited evidence has suggested that LA may contributes to weight control, our study does not support this claim. The primary outcomes assessed were body weight, blood lipids and BMI, which significantly influence obesity. However, in our study, there was no effect on body weight and BMI were observed. This lack of effect may be due to the possibility that excessive doses of linolenic acid could significantly impact appetite, leading to higher calorie intake, which would be counter-productive for weight management.

To the best of our knowledge, this study represents the first randomized controlled trial that compares the efficacy of two distinct lower n-6/n-3 interventions, i.e., 1/2.5 and 2.5/1, in hyperlipidemic patients as a means to regulate lipid metabolism in this population. In our investigation, we compared MP group with LP group in pairs. Notably, the improvement within blood lipids in the LP group did not become more pronounced as the n-6/n-3 ratio decreased when compared to the enhancement observed in the MP group. No significant differences emerged in blood lipid parameters between the MP and LP groups. Serum TC, TG, and LDL levels were decreased in the MP group, while the favorable HDL level increased. Previous research has similarly reported that a lower n-6/n-3 PUFA ratio is not necessarily superior (35) and that the “optimal ratio” of LA and ALA may not represent the most accurate measure of PUFA balance (36). It has been suggested that PUFAs is intake is more effective means of achieving dietary n-6 and n-3 PUFAs balance than simply focusing on proportions (37). The World Health Organization (WHO) also posits that, provided unsaturated fatty acids (USFA) fall within the recommended range, there are no well-founded recommendations for the n-6 to n-3 PUFA or LA to ALA ratios (38). In this context, individual doses of n-6 and n-3 PUFAs should be carefully considered alongside their proportions. In conclusion, both the MP and LP groups demonstrated improved lipid levels, and reducing the n6/n3 ratio proved beneficial for enhancing cardiometabolic health and decreasing the risk of CVD.

To ensure that participants adhered to the required daily cooking oil consumption, we supplied them with specialized oil pots featuring clearly marked scales. This facilitated facilitate control over the quantity of oil utilized during cooking and provide participants with a clear understanding of their daily oil usage, which is a distinguishing aspect of our trial. Furthermore, we acknowledge that prior studies needed to address the impact of individual compliance on experimental outcomes, as they were population-based investigations. Thus, enhance participant compliance during the intervention phase of our trial, we conducted weekly telephone follow-ups while allowing for unrestricted eating. Health education was provided to inform the patients about disease-related knowledge, such as dietary precautions, and to understand their daily eating habits. However, we did not impose restrictions on participants’ energy intake or food types were not restricted. Regular follow-ups and health guidance ensured participants’ compliance, rendering the regulatory effects of n-3 PUFA on blood lipids and body weight more persuasive.

It is important to note that our evaluation of quality of life serves as a reliable indicator of lipid-regulating therapy efficacy in hyperlipidemic patients, and holds significant value in guiding subsequent treatment. We employed the SF-36 quality of life scale to assess changes in participants’ physical function, bodily pain, social function, and other aspects before and after the intervention. Our findings revealed no differences in scores across all p aspects among participants prior to the intervention. However, following the 2-month perilla oil intervention significant between-group differences in energy and total score were observed in the experimental group. In both the MP and LP groups, scores for physical function, vitality, social function, and mental health were significantly improved after the intervention. A possible explanation is that reducing the n-6/n-3 ratio can enhance patients’ blood lipid levels, regulate neurological function and mood, and mitigate the detrimental effects of negative emotions on the disease. Consequently, the quality of life scores for the MP and LP groups improved post- intervention. Other studies have also shown that change dietary changes can reduced blood lipids and body weight while improving mood in hyperlipidemic patients. Although this study did not involve a comprehensive dietary pattern in this study, the rich linolenic acid in perilla oil increased the intake of n-3 PUFAs intake and adjusted the proportion of dietary fatty acids in the diet, further substantiating the aforementioned conclusions.

Oil is a fundamental component of the diet, with its nutritional value determined by both the quantity of oil consumed and the composition of FAs within it. The edible mixed oils, created by blending various fats in specific proportions, address the limitations of single-source fats, which may lack certain nutritional elements. Mixed oils not only satisfy the body’s requirement for various fatty acids but also enhance the flavor of fats. Different fats contain distinct fatty acids, with varying ratios of SFA, monounsaturated fatty acids (MUFA), and PUFA, which impact their nutritional effects. Research has demonstrated that soybean oil is abundant in PUFAs and phytosterols, which can lower blood lipids and protect against cardiovascular and cerebrovascular diseases. However, the insufficient oleic acid content in soybean oil leads to an imperfect nutritional profile. Flaxseed oil, vegetable oil, is rich in n-3 PUFA components and vitamin E, offering antioxidant, anti-inflammatory, and anti-cancer properties. Additionally, flaxseed oil shows considerable potential in preventing CVD and treating diabetes and neurological disorders. Since single-oil nutrition is inadequate to meet the demand for nutritious and healthy oil, a more balanced fatty acid composition is needed for cooking oil. Notably, the distinct fishy taste of deep-sea fish oil can negatively affect the flavor of cooking oil, while flaxseed oil, another vegetable oil, also contains ample n-3 PUFA. Consequently, in this study utilized soybean oil and ALA-rich perilla oil as base oils to prepare the relevant oils. Our findings revealed that both the MP and LP groups experienced similar improvements in blood lipids and quality of life. However, the LP group required more perilla oil, resulting in higher production costs compared to the MP group, potentially posing a financial burden for consumers seeking to increase their ALA intake. Furthermore, the greater perilla oil content in the LP group influenced its flavor and taste. This research can serve as a theoretical foundation for the development and application of nutritionally balanced oil blends.

## 5. Conclusion

Adopting a dietary habit with a lower n-6/n-3 ratio dietary habit positively impacts lipid parameters and quality of life for individuals with hyperlipidemia. Furthermore, it has the potential to enhances patients’ cardiometabolic health and provides protection against CVD. Importantly, excessively reducing the n6/n3 ratio does not lead to additional improvements in blood lipids. These findings reveal an alternative approach for reducing total cholesterol and triglycerides while simultaneously increasing HDL-c level. To substantiate the results of this study, further research involving larger samples is required. Moreover, a deeper exploration of the mechanisms by which different n-6/n-3 ratios influence lipid regulation would be an intriguing subject for future investigations.

## Data availability statement

The original contributions presented in the study are included in the article, further inquiries can be directed to the corresponding authors.

## Ethics statement

The studies involving human participants were reviewed and approved by the ethical review board of the Affiliated Hospital of Jiangnan University. The patients/participants provided their written informed consent to participate in this study.

## Author contributions

YY was responsible for the operation of the experiment, data collection, data interpretation, and writing and revision of the manuscript, under the direction and assistance of BZ, YW, and FZ, who assisted with each step in the study and completion of the manuscript. YX and DL assisted in the completion of the experiment. JiY, HC, and JS were in charge of grouping and followed-up on the trial. All authors have read and agreed to the published version of the manuscript.

## Funding

This work was supported by National Natural Science Foundation of China (grant numbers 32101033), the National Key Research and Development Program of China (2022YFF1100601), the Natural Science Foundation of Jiangsu Province (grant numbers BK20210060, BK20210468) and Soft Science Project of Wuxi Science and Technology Association (KX-22-A02, KX-22-C136).

## Conflict of interest

YX, DL, JiY, JuY, JS, HC, YW, and FZ are employed by Yixing Institute of Food and Biotechnology Co., Ltd.

The authors declare that the research was conducted in the absence of any commercial or financial relationships that could be construed as a potential conflict of interest.

## Publisher’s note

All claims expressed in this article are solely those of the authors and do not necessarily represent those of their affiliated organizations, or those of the publisher, the editors and the reviewers. Any product that may be evaluated in this article, or claim that may be made by its manufacturer, is not guaranteed or endorsed by the publisher.

## Figures and Tables

**Table:** 

CVD	Cardiovascular disease
BMI	Body mass index
FA	Fatty acid
ALA	Alpha-linolenic acid
LA	Linolenic acid
AA	Arachidonic acid
MUFA	Monounsaturated fatty acids
PUFA	Polyunsaturated fatty acids
SFA	Saturated fatty acids
USFA	Unsaturated fatty acids
FM	Fat mass
WC	Waist circumference
WHR	Waist-hip ratio
TC	Total cholesterol
TG	Total triglyceride
HDL-c	High- density lipoprotein cholesterol
LDL-c	Low-density lipoprotein cholesterol

## References

[1] Chen H-J Yan X-Y Sun A Zhang L Zhang J Yan Y-E. Adipose extracellular matrix deposition is an indicator of obesity and metabolic disorders. J Nutr Biochem. (2023) 111:109159. doi: 10.1016/j.jnutbio.2022.109159, PMID: 36162565

[2] Landmesser U Poller W Tsimikas S Most P Paneni F Lüscher TF. From traditional pharmacological towards nucleic acid-based therapies for cardiovascular diseases. Eur Heart J. (2020) 41:3884–99. doi: 10.1093/eurheartj/ehaa229, PMID: 32350510

[3] Soppert J Lehrke M Marx N Jankowski J Noels H. Lipoproteins and lipids in cardiovascular disease: from mechanistic insights to therapeutic targeting. Adv Drug Deliv Rev. (2020) 159:4–33. doi: 10.1016/j.addr.2020.07.019, PMID: 32730849

[4] Powell-Wiley TM Poirier P Burke LE Després J-P Gordon-Larsen P Lavie CJ. Obesity and cardiovascular disease: a scientific statement from the American Heart Association. Circulation. (2021) 143:e984–e1010. doi: 10.1161/CIR.0000000000000973, PMID: 33882682 PMC8493650

[5] Karr S. Epidemiology and management of hyperlipidemia. Am J Manag Care. (2017) 23:S139–48. PMID: 28978219

[6] Navar-Boggan AM Peterson ED D'Agostino RB Neely B Sniderman AD Pencina MJ. Hyperlipidemia in early adulthood increases long-term risk of coronary heart disease. Circulation. (2015) 131:451–8. doi: 10.1161/CIRCULATIONAHA.114.012477, PMID: 25623155 PMC4370230

[7] Djuricic I Calder PC. Pros and cons of long-chain Omega-3 polyunsaturated fatty acids in cardiovascular health. Annu Rev Pharmacol Toxicol. (2023) 63:383–406. doi: 10.1146/annurev-pharmtox-051921-090208, PMID: 36662586

[8] Lai HT de Oliveira Otto MC Lemaitre RN McKnight B Song X King IB. Serial circulating omega 3 polyunsaturated fatty acids and healthy ageing among older adults in the cardiovascular health study: prospective cohort study. BMJ. (2018) 363:k4067. doi: 10.1136/bmj.k4067, PMID: 30333104 PMC6191654

[9] Iggman D Ärnlöv J Cederholm T Risérus U. Association of Adipose Tissue Fatty Acids with Cardiovascular and all-Cause Mortality in elderly men. JAMA Cardiol. (2016) 1:745–53. doi: 10.1001/jamacardio.2016.2259, PMID: 27541681

[10] Davinelli S Intrieri M Corbi G Scapagnini G. Metabolic indices of polyunsaturated fatty acids: current evidence, research controversies, and clinical utility. Crit Rev Food Sci Nutr. (2021) 61:259–74. doi: 10.1080/10408398.2020.1724871, PMID: 32056443

[11] Park S Lee J-J Lee J Lee JK Byun J Kim I. Lowering −6/−3 ratio as an important dietary intervention to prevent LPS-inducible dyslipidemia and hepatic abnormalities in mice. Int J Mol Sci. (2022) 23:6384. doi: 10.3390/ijms23126384, PMID: 35742829 PMC9224551

[12] Galli C Risé P Sirtori C. Eicosapentaenoic acid for prevention of major coronary events. Lancet. (2007) 370:215. doi: 10.1016/S0140-6736(07)61113-1, PMID: 17658383

[13] Bhatt DL Steg PG Miller M Brinton EA Jacobson TA Ketchum SB. Cardiovascular risk reduction with Icosapent ethyl for hypertriglyceridemia. N Engl J Med. (2019) 380:11–22. doi: 10.1056/NEJMoa1812792, PMID: 30415628

[14] Nicholls SJ Lincoff AM Garcia M Bash D Ballantyne CM Barter PJ. Effect of high-dose Omega-3 fatty acids vs corn oil on major adverse cardiovascular events in patients at high cardiovascular risk: the STRENGTH randomized clinical trial. JAMA. (2020) 324:2268–80. doi: 10.1001/jama.2020.22258, PMID: 33190147 PMC7667577

[15] Abdelhamid AS Brown TJ Brainard JS Biswas P Thorpe GC Moore HJ. Omega-3 fatty acids for the primary and secondary prevention of cardiovascular disease. Cochrane Database Syst Rev. (2018) 11:CD003177. doi: 10.1002/14651858.CD003177.pub4, PMID: 30521670 PMC6517311

[16] Simopoulos AP. An increase in the Omega-6/Omega-3 fatty acid ratio increases the risk for obesity. Nutrients. (2016) 8:128. doi: 10.3390/nu8030128, PMID: 26950145 PMC4808858

[17] Schulze MB Minihane AM Saleh RNM Risérus U. Intake and metabolism of omega-3 and omega-6 polyunsaturated fatty acids: nutritional implications for cardiometabolic diseases. Lancet Diabetes Endocrinol. (2020) 8:915–30. doi: 10.1016/S2213-8587(20)30148-0, PMID: 32949497

[18] Scaioli E Liverani E Belluzzi A. The imbalance between n-6/n-3 polyunsaturated fatty acids and inflammatory bowel disease: a comprehensive review and future therapeutic perspectives. Int J Mol Sci. (2017) 18:2619. doi: 10.3390/ijms18122619, PMID: 29206211 PMC5751222

[19] Van Elst K Brouwers JF Merkens JE Broekhoven MH Birtoli B Helms JB. Chronic dietary changes in n-6/n-3 polyunsaturated fatty acid ratios cause developmental delay and reduce social interest in mice. Eur Neuropsychopharmacol. (2019) 29:16–31. doi: 10.1016/j.euroneuro.2018.11.1106, PMID: 30563719

[20] Mozaffarian D. Dietary and policy priorities for cardiovascular disease, diabetes, and obesity: a comprehensive review. Circulation. (2016) 133:187–225. doi: 10.1161/CIRCULATIONAHA.115.018585, PMID: 26746178 PMC4814348

[21] Billingsley HE Hummel SL Carbone S. The role of diet and nutrition in heart failure: a state-of-the-art narrative review. Prog Cardiovasc Dis. (2020) 63:538–51. doi: 10.1016/j.pcad.2020.08.004, PMID: 32798501 PMC7686142

[22] Barkas F Nomikos T Liberopoulos E Panagiotakos D. Diet and cardiovascular disease risk among individuals with familial hypercholesterolemia: systematic review and Meta-analysis. Nutrients. (2020) 12:2436. doi: 10.3390/nu12082436, PMID: 32823643 PMC7468930

[23] Huang J Chen C Song Z Chang M Yao L Jin Q. Effect of microwave pretreatment of perilla seeds on minor bioactive components content and oxidative stability of oil. Food Chem. (2022) 388:133010. doi: 10.1016/j.foodchem.2022.133010, PMID: 35468463

[24] Adam G Robu S Flutur M-M Cioanca O Vasilache I-A Adam A-M. Applications of *Perilla frutescens* extracts in clinical practice. Antioxidants. (2023) 12:727. doi: 10.3390/antiox12030727, PMID: 36978975 PMC10045045

[25] Li HZ Zhang ZJ Hou TY Li XJ Chen T. Optimization of ultrasound-assisted hexane extraction of perilla oil using response surface methodology. Ind Crop Prod. (2015) 76:18–24. doi: 10.1016/j.indcrop.2015.06.021

[26] Ahmed HM. Ethnomedicinal, phytochemical and pharmacological investigations of (L.) Britt. Molecules. (2018) 24:102. doi: 10.3390/molecules24010102, PMID: 30597896 PMC6337106

[27] Koonyosying P Kusirisin W Kusirisin P Kasempitakpong B Sermpanich N Tinpovong B. Perilla fruit oil-fortified soybean milk intake alters levels of serum triglycerides and antioxidant status, and influences phagocytotic activity among healthy subjects: a randomized placebo-controlled trial. Nutrients. (2022) 14:1721. doi: 10.3390/nu14091721, PMID: 35565689 PMC9103900

[28] Hashimoto M Matsuzaki K Maruyama K Sumiyoshi E Hossain S Wakatsuki H. *Perilla frutescens* seed oil combined with *Anredera cordifolia* leaf powder attenuates age-related cognitive decline by reducing serum triglyceride and glucose levels in healthy elderly Japanese individuals: a possible supplement for brain health. Food Funct. (2022) 13:7226–39. doi: 10.1039/D2FO00723A 35722977

[29] Noerman S Landberg R. Blood metabolite profiles linking dietary patterns with health-toward precision nutrition. J Intern Med. (2022) 293:408–32. doi: 10.1111/joim.13596, PMID: 36484466

[30] Paul S Smith AAT Culham K Gunawan KA Weir JM Cinel MA. Shark liver oil supplementation enriches endogenous plasmalogens and reduces markers of dyslipidemia and inflammation. J Lipid Res. (2021) 62:100092. doi: 10.1016/j.jlr.2021.100092, PMID: 34146594 PMC8281607

[31] Ware JE Sherbourne CD. The MOS 36-item short-form health survey (SF-36). I. Conceptual framework and item selection. Med Care. (1992) 30:473–83. doi: 10.1097/00005650-199206000-00002, PMID: 1593914

[32] Ware JE. SF-36 health survey update. Spine. (2000) 25:3130–9. doi: 10.1097/00007632-200012150-00008, PMID: 11124729

[33] Rodriguez D Lavie CJ Elagizi A Milani RV. Update on Omega-3 polyunsaturated fatty acids on cardiovascular health. Nutrients. (2022) 14:5416. doi: 10.3390/nu14235146, PMID: 36501174 PMC9739673

[34] Wilson PWF Polonsky TS Miedema MD Khera A Kosinski AS Kuvin JT. Systematic review for the 2018 AHA/ACC/AACVPR/AAPA/ABC/ACPM/ADA/AGS/APhA/ASPC/NLA/PCNA guideline on the Management of Blood Cholesterol: a report of the American College of Cardiology/American Heart Association task force on clinical practice guidelines. Circulation. (2019) 139:e1144–61. doi: 10.1161/CIR.0000000000000626, PMID: 30586775

[35] Zhang J Wang O Guo Y Wang T Wang S Li G. Effect of increasing doses of linoleic and α-Linolenic acids on high-fructose and high-fat diet induced metabolic syndrome in rats. J Agric Food Chem. (2016) 64:762–72. doi: 10.1021/acs.jafc.5b04715, PMID: 26743332

[36] Harris WS. The omega-6/omega-3 ratio and cardiovascular disease risk: uses and abuses. Curr Atheroscler Rep. (2006) 8:453–9. doi: 10.1007/s11883-006-0019-7, PMID: 17045070

[37] Wijendran V Hayes KC. Dietary n-6 and n-3 fatty acid balance and cardiovascular health. Annu Rev Nutr. (2004) 24:597–615. doi: 10.1146/annurev.nutr.24.012003.132106, PMID: 15189133

[38] Fats and fatty acids in human nutrition. Report of an expert consultation. FAO Food Nutr Pap. (2010) 91:1–166. PMID: 21812367

